# Magnetic Nanoparticles as MRI Contrast Agents

**DOI:** 10.1007/s41061-020-00302-w

**Published:** 2020-05-07

**Authors:** Ashish Avasthi, Carlos Caro, Esther Pozo-Torres, Manuel Pernia Leal, María Luisa García-Martín

**Affiliations:** 1 BIONAND - Centro Andaluz de Nanomedicina y Biotecnología, Junta de Andalucía-Universidad de Málaga, C/Severo Ochoa, 35, 29590 Málaga, Spain; 2 Departamento de Química Orgánica y Farmacéutica, Facultad de Farmacia, Universidad de Sevilla, 41012 Seville, Spain; 3 Networking Research Center on Bioengineering, Biomaterials and Nanomedicine, CIBER-BBN, Málaga, Spain

**Keywords:** Magnetic nanoparticles, Iron oxide nanoparticles, Magnetic resonance imaging, Cancer, Diagnosis

## Abstract

Iron oxide nanoparticles (IONPs) have emerged as a promising alternative to conventional contrast agents (CAs) for magnetic resonance imaging (MRI). They have been extensively investigated as CAs due to their high biocompatibility and excellent magnetic properties. Furthermore, the ease of functionalization of their surfaces with different types of ligands (antibodies, peptides, sugars, etc.) opens up the possibility of carrying out molecular MRI. Thus, IONPs functionalized with epithelial growth factor receptor antibodies, short peptides, like RGD, or aptamers, among others, have been proposed for the diagnosis of various types of cancer, including breast, stomach, colon, kidney, liver or brain cancer. In addition to cancer diagnosis, different types of IONPs have been developed for other applications, such as the detection of brain inflammation or the early diagnosis of thrombosis. This review addresses key aspects in the development of IONPs for MRI applications, namely, synthesis of the inorganic core, functionalization processes to make IONPs biocompatible and also to target them to specific tissues or cells, and finally in vivo studies in animal models, with special emphasis on tumor models.

## Introduction

Magnetic resonance imaging (MRI) is one of the main in vivo imaging modalities, along with positron emission tomography (PET), computed tomography (CT) and ultrasound imaging. MRI is the most versatile of all of these, being able to provide both anatomical and functional information with excellent image quality, and, most importantly, using non-ionizing radiation, which allows longitudinal studies to be performed without the risk of side effects. The MRI signal comes from the radiofrequency signal of protons magnetized by an external magnetic field. These protons originate mainly from water molecules. The application of radiofrequency pulses is used to excite the magnetization, and magnetic field gradients are used to provide spatial localization. Contrast in MRI reflects differences in signal intensity, which depends on the concentration of water molecules within the tissue, the relaxation times, T_1_ and T_2_, of the water protons and the mobility of the water molecules (diffusion, flow) [1]. Additionally, image contrast can be further enhanced using contrast agents (CAs), with Gd-chelates being used most commonly in clinical practice. However, CAs lack specificity and have recently been related to toxicity issues caused by the unexpected release of free Gd. Magnetic nanoparticles have emerged as a promising alterative with improved properties in terms of specificity and biocompatibility. Over the past two decades, many studies have aimed at the development of new magnetic nanomaterials that can serve to improve the diagnosis and treatment of many different diseases. Among these nanomaterials, iron oxide nanoparticles (IONPs) have been investigated most extensively as CAs for MRI due to their magnetic properties, that is, the superparamagnetism that leads to very high relaxivity, their high biocompatibility, since they can be incorporated into iron metabolism, and also the easy functionalization of their surfaces with target molecules for molecular imaging purposes [2].

The first step in the development of IONPs is synthesis of the magnetic core, for which many different methods have been proposed, all aiming at strict control of the size, shape and magnetic properties, so that the synthesis process can be performed under highly reproducible conditions, which is one of the essential requirements for the potential clinical translation of these new nanomaterials [3]. Functionalization of magnetic nanoparticles is then needed to make them soluble in aqueous media and to provide them with stability and biocompatibility [4]. Further functionalization may include the addition of different molecules to target specific tissues or cells [5]. The most relevant functionalization strategies will be discussed in detail in this review. Finally, the in vivo characterization of IONPs is the most critical aspect in the development of IONPs for biomedical applications. Although many new nanomaterials show excellent in vitro properties, most of them fail when tested in vivo. Thus, around 6500 studies (PubMed database) on magnetic nanoparticles have been published since 2010, in which IONPs often appear as promising new CAs for MRI. However, up to now, extremely low clinical translation has been achieved [6]. Therefore, comprehensive studies with appropriate in vivo experimental models are of paramount importance for the successful development and eventual clinical translation of these nanomaterials.

In this review, we describe the recent advances in regard to the synthesis, functionalization and in vivo applications of IONPs as MRI CAs for the diagnosis of several pathologies, with special emphasis on cancer diagnosis.

## Methods for the Synthesis of IONPs

**Fig. 1:**
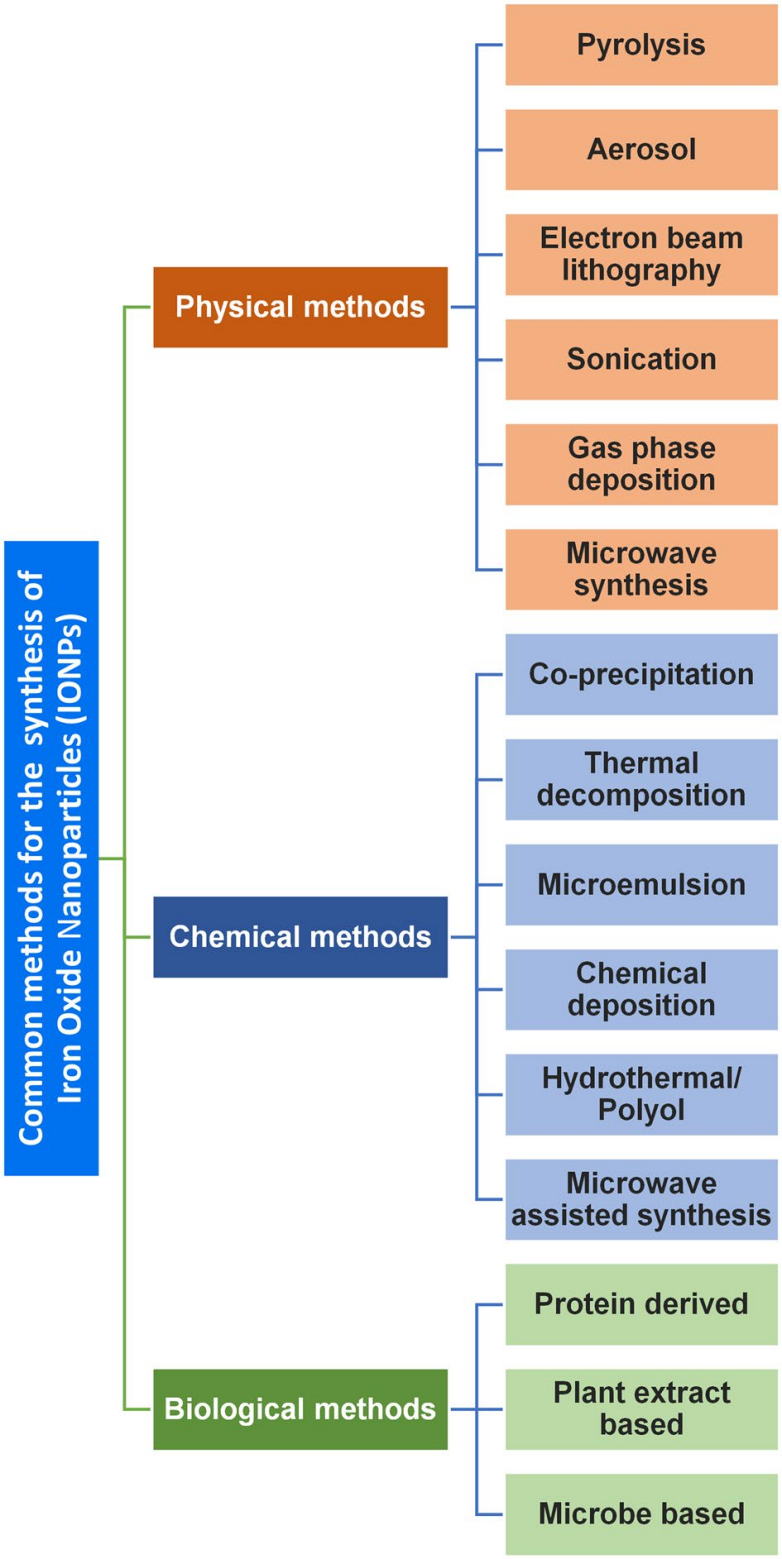
Methods used in the synthesis of iron oxide nanoparticles (IONPs)

Over the past few decades, various procedures to synthesize IONPs have come to fruition. The ultimate goal of these procedures is to gain complete control over the properties of IONPs, such as size, shape, saturation magnetization, etc. However, this has not yet been achieved completely. The main hindrance behind this failure is the inability to fully determine the science behind the processes and their mutual interactions, but it is not so distant in the future that we will be successful. Figure 1 shows different methods to synthesize IONPs, which are described in detail below, along with their pros and cons.

### Coprecipitation

**Fig. 2:**
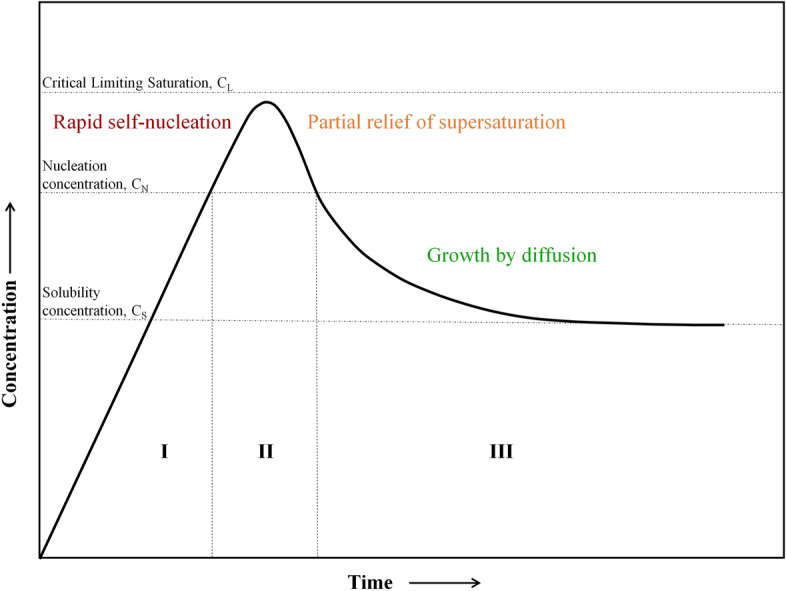
LaMer’s model depicting the nucleation and growth process of the nanoparticles. Adapted with permission from [10]. Copyright (1950) American Chemical Society

Coprecipitation is the method most commonly used for the synthesis of IONPs due to its facile nature. Massart [7] pioneered the existing scientific knowledge established by Le Fort [8] and Elmore [9] regarding the synthesis of magnetic colloids, and stressed the importance of the stoichiometric ratio between Fe(II):Fe(III) being 1:2. The synthesis process described by Massart requires the addition of alkaline medium (pH ~ 11, slowly or rapidly) into the iron salts solution at room temperature or at elevated temperature. This mixture requires an inert atmosphere to prevent nanoparticles from oxidizing. It was later established that the synthesis of particles follows the LaMer’s model of nucleation and growth [10] (Fig. 2). The synthesis process has been described to occur in two steps, as shown below [11–14]

$${\text{Fe}}^{{{2} + }} + {\text{ 2Fe}}^{{{3} + }} + {\text{ 8OH}}^{ - } { \leftrightarrows } {\text{Fe}}\left( {{\text{OH}}} \right)_{{2}} + {\text{ 2Fe}}\left( {{\text{OH}}} \right)_{{3}} \to {\text{ Fe}}_{{3}} {\text{O}}_{{4}} \downarrow + {\text{ 4H}}_{{2}} {\text{O}}.$$

However, Lagrow et al*.* [15] recently challenged this mechanism of synthesis. They claimed that while increasing the pH via sodium carbonate, two intermediate phases are formed, one poorly crystalline ferrihydrite and another crystalline iron hydroxide carbonate. This ferrihydrite eventually grows into iron oxide at the cost of iron hydroxy carbonate. Even though Lagrow’s proposed mechanism seems to answer a few loopholes undescribed by Massart, improving the homogeneity and reproducibility of the nanoparticles, it fails to ascertain if the same mechanism is followed when ammonia or ammonium hydroxide is used.

Irrespective of the mechanism followed, nucleation is judged as the size-determining step and is exploited to modulate the size of particles [14–16]. The nature of particles depends on various other factors, such as the type of salts used (e.g. chlorides, sulfates, nitrates, perchlorates, etc.), the Fe^2+^ and Fe^3+^ ratio, pH and the ionic strength of the media, along with the reaction environment [17–30]. Jiang et al. [24] showed that the particle size distribution is narrowed if the homogeneity of pH within the solution is improved by adding urea to the reaction mixture. There are also reports suggesting that particle size decreases with increasing pH [17]. A similar trend is observed between particle stability and iron concentration, but substantial studies are lacking to support this observation [23]. Particles with different morphologies, such as nanodots, ellipsoid, spherical, clusters or necklace like, can be synthesized by varying their aging conditions [25–27]. Itoh et al. [26] synthesized ellipsoidal and spherical hematite nanoparticles by aging them in phosphate ions and nitriloacetic acid (NTA), respectively. The relationship between shape/size and the electrostatic surface density of particles is linked to the interfacial tension between the oxide and the solution, which causes a decrease in the surface energy, thus modulating shape and size [28]. If a modern method like ultrasonication is used with coprecipitation, it can yield narrowly distributed particles, as shown by Bui et al. [31], who compared their modified version of the coprecipitation method (using ultrasonication instead of stirring) to the solvothermal method, and found the former to yield more homogeneous and small sized nanoparticles. However, the comparison between their method and the conventional coprecipitation method (with stirring) is missing. The major advantages of the coprecipitation method are its time saving facile nature, with no requirement of high temperature or pressure, and the production of particles with high yield and easily scalable to large quantities. However, the particles synthesized with this method generally lack homogeneity and form single and also multicore nanoparticles. Particles thus synthesized also tend to form aggregates, which leads to an undesired assortment of blocking temperatures. Another disadvantage of this method is that the pH of the resultant solution is too high, thus requiring neutralization before they can be used for biological applications.

### Thermal Decomposition

**Fig. 3:**
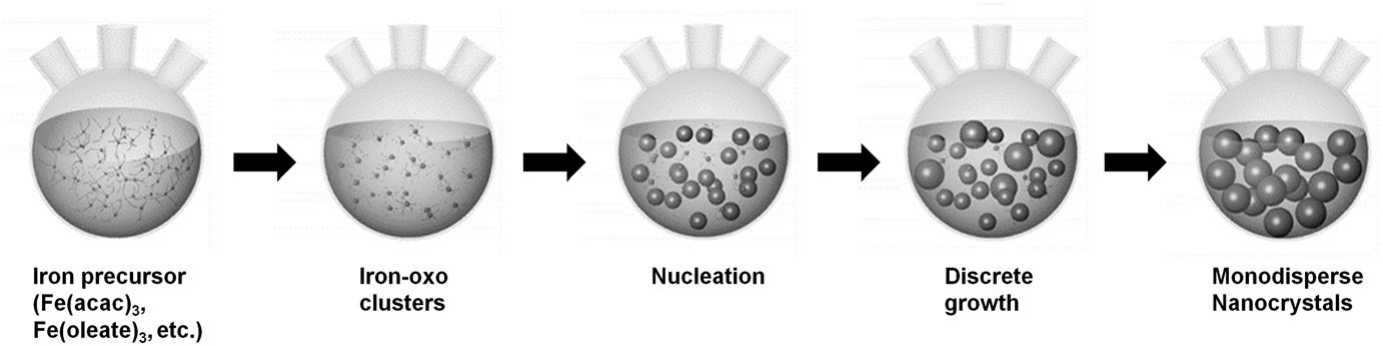
Different stages during the synthesis of IONPs in the thermal decomposition method. Adapted and modified with permission from [41]. Copyright (2013) American Chemical Society

In this method of synthesis, high temperatures are exploited to break down the precursor to yield nuclei as well as their further growth into nanoparticles (Fig. 3). It started as a way to ease the study of properties of systems with narrow size distribution [32]. Smith and Wychlk were among the first researchers who utilized this method to synthesize colloidal dispersions of iron using iron pentacarbonyl [Fe(CO)_5_] as a precursor, along with different solvents and the addition of different polymers. They concluded that the polymers added during the reaction not only coated the dispersions forming stable particles, but also acted as catalysts for the decomposition [33, 34]. They suggested that the decomposition takes place at 140–160 ℃ in the presence of butadiene polymers while gathering support from the mechanistic studies conducted by Bergman and coworkers [35]. Later, their hypothesis was verified experimentally, showing the presence of an intermediate carbonyl complex formed after decomposition of Fe(CO)_5_ [36]. The reaction takes place in two main steps: nucleation and growth. This separation of stages can be used advantageously to alter the size and shape of nanoparticles as demonstrated by Hyeon et al*.* [37] and Jana et al*.* [38]. They used iron oleate as precursor and proposed that nucleation starts at 200–240 ℃, initiated by dissociation of one of the three oleates available in one molecule of iron oleate [Fe-(oleate)_3_], while the growth begins at 300 ℃ with the subsequent dissociation of the remaining two oleates. The complete mechanism of the reaction is not fully understood even though it has been widely studied, both experimentally and computationally [39–41]. Nonetheless, these studies led to the discovery of “polyiron oxo clusters” species as the actual precursor for the formation of nanoparticles, as initially suggested by Wells [36]. More recent studies have reported the synthesis of a new precursor by synthesizing an intermediate between Fe(CO)_*x*_ and oleylamine (OLA), and achieved controllable size of 2.3–10 nm [42].

To date, different precursors have been reported in the literature: iron acetylacetonate [Fe(acac)_3_] [43], iron cupferron [Fe(cup)] [44], iron chloride (FeCl_3_) [45], iron pentacarbonyl [Fe(CO)_5_] [46], along with different iron complexes such as iron oleate [45], iron stearate [38] and iron eruciate [47]. Depending on the process involved and the size required, it becomes important to select the right precursor as the reaction proceeds differently depending on the way the precursor is broken down [48].

There are several other factors that could affect the size and morphology of particles, such as temperature, nature of the solvent, reactants ratio, reflux time, and seed concentration [45, 49, 50]. Thus, Hyeon found that the heating rate of the reaction, along with the boiling point of the solvent used, is also a crucial factor to adjust the size of the nanoparticles [45], and Pellegrino’s group concluded that there is an inverse relationship between the size of the nanoparticles and the heating rate [49]. However, controversy still exists regarding the role of the temperature ramp in the synthesis of IONPs, and, therefore, comprehensive and deeper studies are still needed to properly elucidate the mechanism involved.

Kovalenko et al. [51] showed the importance of surfactants, not only to prevent aggregation, but also to modulate shape and size. They displayed the use of fatty acids, such as oleic acid (OA) or salts of OA, to synthesize spheres and cubic nanoparticles, respectively. Later, several groups have tried to shed light on the role of OA as well as other fatty acids regarding the size and shape of IONPs, but up to now, a fully elucidated theory is still lacking [52–59]. Quality of particles can be further improved by the controlled addition of water and oxygen in the inert environment to decrease crystal defects, and improve magnetic properties and homogeneity [60, 61].

In summary, thermal decomposition, albeit a bit complex and time-consuming, yields very homogenous and monodisperse nanoparticles, making it one of the most used methods to synthesize nanoparticles for biological applications. The shape and size of nanoparticles can be controlled by tuning the parameters described above. Major drawbacks of this method include the inability to properly scale up and the lack of dispersibility of the particles in aqueous solvents, although this can be remedied by surface modifications in situ, as described by Li et al*.* [56, 62], or using post preparative methods, as explained in greater detail in later sections of this review.

### Hydrothermal and Solvothermal Synthesis

In this method, the hydrolysis and oxidation (or neutralization) reaction takes place in a reactor or autoclave at high temperature and pressure. Depending on the reaction solvent, it is either referred to as hydrothermal (if the solvent is water) or solvothermal (any other solvent or combination). Both reactions follow the aforementioned model of nucleation and growth [63, 64]. There have been several reports [4, 37, 65–68] on the use of this method to synthesize magnetic nanoparticles as well as its comparison with other methods [69].

The reaction parameters, such as temperature, reactor size, time, concentration of the reactants, and the nature of the solvent and capping agents, affect the size, shape and other properties of the final product. Out of all these parameters, the effect of the solvent has been studied the most [70, 71], closely followed by that of the surfactant [72, 73]. The particles show a preferential surface binding towards the carboxylate from the OA rather than the amine from the oleylamine [72], which very likely is the case for every method described in this article, although it still needs verification. This preferential binding was recently used by Brewster et al. [73] to present a new way to control the particle size and crystal phase. They varied the carbon chain length in the iron carboxylate, which was used as the precursor, and showcased the effect of two different ligands, amine and carboxylic acid, which were added to the reaction [73]. They demonstrated that the size of the particles decreased as the carboxylate chain length increased in the presence of amine ligands, while no definite trend was observed when varying the carboxylate free ligands.

The hydrothermal/solvothermal method has also been used to synthesize other ferrites [74]. Kim et al. [75] recently demonstrated a gram scale yield of magnetite nanoclusters by modifying the procedure and utilizing trisodium dihydrate, but, to the best of our knowledge, this is the only report for large scale synthesis using this method. To further exploit the particles thus formed for biological applications, surface coating becomes necessary, as will be discussed in detail in the subsequent section. Polymers such as polyvinylpyrrolidone (PVP), polyacrylic acid (PAA) and polyethanolimine (PEI), have been shown to improve the magnetic properties when used in the synthesis of monodispersed clusters [76]. Recently, Köçkar et al. [77] explained a way to get in-situ capping of IONPs with tartaric acid/ascorbic acid/mixture of two, which led to the synthesis of uniform, un-agglomerated, biocompatible particles of less than 8 nm with good saturation magnetization. The hydrothermal/solvothermal method is, therefore, an ideal method for the synthesis of iron oxide nanoparticles, mainly nanoclusters. However, the main disadvantage of this method is that, due to the lack of stirring inside the autoclave, monodispersity, as well as scalability, can sometimes be hindered.

### Polyol Method

This method is an iteration of the solvothermal method, with polyols being used as solvents to synthesize nanoparticles by dissolving the precursor, solubilizing in the diol at high temperatures, and eventually leading to the formation of metal nuclei and particles. Following previous works pertaining to synthesis of metallic powders [78–84], Caruntu et al*.* described this method to synthesize nanocrystalline metal oxide nanoparticles by synthesizing magnetite nanoparticles [85]. They explained the mechanism stating that reduction starts from the liquid state rather than the solid, and the nanoparticles are formed in two steps: hydroxides are formed first and then metal centers are chelated. Heterogeneous nucleation performs better than homogeneous nucleation as it has been studied to provide a better separation between nucleation and growth, thus giving better control over the size, shape and crystallinity [78]. Polyols play multiple roles, acting as reducing agent, stabilizer and solvent [86], modulating the process to yield large and small clusters [87], nanoparticles [88] or single-core/multicore nanoparticles [89]. Different polyols have been exploited for the synthesis of iron oxide nanoparticles, such as diethylene glycol, giving 3 nm particles [90], or triethylene glycol, giving 10 nm particles [91]. However, Cai et al. [92] reported that only triethylene glycol gives non-aggregated nanoparticles. To our knowledge, there are no reports on the use of tetra or penta ethylene glycol, which could have ameliorated the agglomeration problem even more, if the trend described holds to be true. Other parameters that have been identified to modulate the size, shape, crystallinity and saturation magnetization are temperature, time, precursor concentration, and surfactant. The role of water was studied by Hemery et al. [93], when its importance was revealed by inability of the anhydrous iron chloride to produce magnetic particles [93]. The impact of stoichiometry in polyol synthesis has been studied by Wetegrove et al. [94], showing that the increase in Fe^3+^ concentration forms larger crystallites and the increase in Fe^2+^ content promotes nucleation [94].

As stated above, hydrophilicity is important, which is generally lacking in particles synthesized using the polyol method. However, there are reports of the synthesis of hydrophilic nanoparticles using this method [88, 95] but their limitations include the lack of a surface functionality for bioconjugation. This problem has further been remedied by the use of polyamines [96], polyimine with polyol [97], polyamine with polyol [98] and PAA [99]. The research done by Babić-Stojić et al. [100], wherein they esterified 3 nm IONPs in situ, implied the importance of the surface layer in the properties of nanoparticles.

The morphology of the particles is of equal importance as size in in vivo applications and has been shown to be altered by the addition of halide ions [101]. There have also been advancements in solvents, such as the thermostable ionic solvent [P6,6,6,14][Tf2N], which has been shown to be capable of synthesizing quasi spherical magnetite nanoparticles of around 14 nm [102].

In conclusion, the method described herein has the advantage of being environment friendly, scalable, and good for synthesizing both single and multicore particles. However, it has the drawback that the particles thus formed lack homogeneity.

### Sol–Gel Method

This is a two-step chemical method, with the first step being the synthesis of the sol (particles in a solution) via hydroxylation of the precursors, and the second step, the formation of a gel by condensation and polymerization. Eventually, heat treatments are used to achieve a proper crystalline state. Costa et al. [19] were among the first to synthesize magnetic nanoparticles using this method, but they failed to identify the correct mechanism. Subsequently, the work of Portugal et al. [103], made the mechanism a bit clearer upon finding signatures of iron hydroxide, but the exact mechanism is still unknown. Like in the polyol method, the solvent is shown to affect the ferrite grain as well, but changes in grain size have been attributed to a different growth model with two different solvents [104]. Water concentration is also shown to improve hardness and structural defects [105].

Size and shape are also affected by other parameters such as solvent ratio, time, pH, stirring, gelating agent and, temperature. Liu et al. [106] used different calcination temperatures to synthesize different phases of IONPs, and this transformation has been attributed to two separate mechanisms, crystal regrowth and chemisorption, depending on the temperature. Akbar et al. claimed to have synthesized three different phases of iron oxide (α-Fe_2_O_3_, γ-Fe_2_O_3_, and Fe_3_O_4_) simply by varying the precursor to solvent ratio, thus suggesting the importance of that ratio [107]. The particles were shown to possess higher saturation magnetization. They also observed differences in hematite particle size and morphology when using different precursors, with iron acetate giving rise to smaller spherical particles, while iron nitrate led to larger, quasi cubic particles. These differences were due to the water content as well as the presence of nitrate and carboxylate in the precursors [108]. More recently, Hu et al. [109] reported a new explosion-assisted sol–gel method in which they used ferric nitrate as precursor and citric acid as chelating agent to form a gel. The gel was then homogenized and heated with picric acid to attain highly pure, well dispersed and crystallized magnetite nanoparticles ranging from 3 to 20 nm. The synthesis was proposed to be resulting from the combined action of the complexing of citric acid with metal ions, and the explosion, thus explaining the important role of citric acid, not only as a carbon source, but also to allow the combustion and reduction of the dried gel simultaneously. The chemistry of the sol–gel method is vast, with the involvement of different precursors, gelators as well as chelators, but it is beyond the scope of this review. It is, however, nicely explained by Danks et al. [110].

This method is more recommended for synthesizing thin films [111] and nanocomposites [112, 113] since it can form thin films in just 2 min if the heating source is changed to microwaves, and pure phases can be formed by using high microwave power (600–800 W).

### Microemulsion Method

The microemulsion method is a form of coprecipitation performed in a confined space such as micelles. It generally involves two immiscible liquids with surfactants forming the interfacial layer [114], and is classified as either the water-in-oil method or oil-in-water method [115]. Inouye et al. [116] were the first to report the synthesis of magnetic particles using this method, exploiting the faster oxidation of ferrous ions in micelles.

In water-in-oil microemulsion, a hydrophobic phase is used with aqueous droplets separated by a surfactant [117]. The most common surfactants used are PVP and cetyltrimethylammonium bromide (CTAB). In this method, particles generally collide and coalesce, and break again, leading to the growth of particles, the particle size being determined by the size of the droplets. In a final step, particles are centrifuged and lyophilized to get pure nanoparticles [118–120]. Many articles have been published on the use of this method to synthesize iron oxide nanoparticles [121–124]. Although surfactant concentration is not shown to affect the size, precursor concentration and temperature are important influencers, together with pH [125] and the choice of surfactant [126, 127]. Recently, Singh et al. [128] showed the importance of ionic concentration and temperature on the morphology, size and crystallinity by claiming that, in order to obtain monophasic particles, [Fe^2+^] and [Fe^3+^] should be ≤ 0.09 M and ≤ 0.184 M, respectively, with a temperature range of 65–72 ℃. They also observed changes in the morphology of the particles, from cubes to pentagons to spheres, when increasing the concentration of the surfactant (CTAB) between 0.01 and 0.1 M, but they did not describe the mechanism, or explain why the shape of the CTAB nanodroplets changes upon varying concentration. Nor did they explain why particle size changed with concentration [128]. Bonachhi et al. [129] achieved ultra-small magnetic nanoparticles by using γ-cyclodextrin by hydrolyzing Fe^2+^ ions in aqueous solution, while Lee et al. [130] varied the ratios of the precursor and solvent from 3.6 to 8.1, and achieved 2 to 10 nm magnetite particles. Vidal et al. showed the importance of oleylamine as surfactant to prevent aggregation [131], while Pileni et al. explained the importance of using functionalized surfactants and pH to improve the crystallinity and morphology of the nanoparticles [132]. Following a similar approach, Han et al. used a nonionic surfactant, C_16_E_15_, to synthesize nanoparticles with high saturation magnetization (74.8 emu/g) [133]. It is worth mentioning that if the surfactant described in this method is replaced by a phospholipidic molecule to form particles within liposomes, they are termed magnetoliposomes, which show significantly higher blood half-life [134–136]. However, if the particles are formed within the aqueous compartment, they are known as magnetovesicles. These special particles can be synthesized using film hydration and extrusion [137], sonication [66], phase evaporation [138] and nanoreactor [139], and are very promising for biomedical applications.

Recently, even metallosurfactants have been used as precursors to synthesize particles of around 3 nm [140]. This method has also been utilized in exchanging the capping of iron oxide nanoparticles to improve solubility [141–143].

Similarly, oil-in-water has a hydrophilic solution with oil droplets used as a reactor. Recently, spinel ferrites have been shown to be synthesized using this method, with metal ethylhexanoates as precursors and a pseudo ternary solvent system, which includes oil, surfactant and water in the ratios of 20:20:60 [144]. The oil in water method has also been used as a strategy to cap nanoparticles [145].

The microemulsion method has several advantages, such as providing a narrow range of particles with relative ease, good morphology and without the need for high temperatures. But it also has disadvantages, including scalability, the toxicity of some surfactants, the amount of surfactant used, as well as the need for ligand exchange.

### Aerosol Method

This is also a chemical method, which leads to high production of particles. This method can be subdivided in two categories. The first is spray pyrolysis, in which precursor salts are sprayed into the reactors, where they are condensed and solvent is evaporated, which in turn also means that the size of the particles depends on the droplets [146].

Serna’s group [147] were among the first to synthesize Fe_2_O_3_ nanoparticles using this method. Their study claimed that if small size is the most important feature for the application, iron acetylacetonate should be used because of its exothermic decomposition reaction; however, if crystallinity is to be considered, then iron chloride is favored due to solvent elimination at higher temperature. This leaves other precursor benefits open for exploration. The importance of intraparticle reactions in controlling the size of particles was established later, along with the solvent, rate of evaporation, time spent in the reactor, and temperature. These studies concluded that the heating time and temperature, along with the type of evaporation or reaction taking place during the drying stage, will conform the particle structure as hollow, dense, foam-like, etc. [148, 149]. Zheng et al. [150] recently reported that chloride ions prevent phase transition from γ-Fe_2_O_3_ to α-Fe_2_O_3_ at higher temperatures, leading to higher magnetization, which highlights the importance of chloride ions in the reaction. Das et al. proposed a new strategy to decrease size with high crystallinity by adding ethanol to the ultrasonic pyrolysis [151]. It was explained that the faster evaporation rate of ethanol compared to water, as well as a decrease in surface tension of the water–ethanol solution, led to the formation of smaller droplets and eventually smaller particles. Since the rate of evaporation of the solvent has been stressed and linked to particle size, it might be interesting to see how methanol, or any other solvent with a boiling point lower than that of ethanol, affects the size and crystallinity of particles.

The second category is Laser pyrolysis, a gas phase method that utilizes the heat generated by a laser to heat the precursors and the flow of a gas or a mixture of gases to produce nanoparticles. The sizes of the particles can be controlled by modulating the power of the laser since a direct relationship exists between the two [152, 153]. Zhao et al. [154] were the first to improve on the TEA laser using a cw CO_2_ laser, which yielded particles with higher purity. There have also been reports on use of this method to synthesize hybrid silica-iron oxide composites [155]. Laser pyrolysis has a new iteration, flame spray pyrolysis (FSP), which uses a flame to heat the precursor [156]; the size of the nanoparticles can be controlled by varying the flame length or the oxidant flow rate, and the precursor/fuel composition. Lower flow rate of the oxidant leads to reduced flame length, with higher temperatures thus forming smaller particles and vice versa [157].

The main advantage of this method is that it helps in achieving very high homogeneity and monodispersity irrespective of the complexity of particles, including hybrid silica-iron oxide composites [155].

### Sonochemical Method

This method utilizes acoustic cavitation, which means the formation, growth and collapse of bubbles generated by ultrasound, to synthesize nanoparticles. Instead of using high temperature or pressure directly, this method creates them indirectly by using bubbles or cavities formed in the liquid by the acoustic waves. Further oscillation of such waves helps them gather and store ultrasonic energy, creating a hot spot (~ 5000 K) and leading to the synthesis of particles of different shapes and sizes. This method works for both volatile and non-volatile solvents [158–160]. The reaction medium was already considered the most important factor in controlling the properties of nanoparticles by Suslick et al. [160] when they proposed the method, since the bubbles formed will depend on the vapor pressure of the media. The nature of the particles can also be altered by changing the ultrasonic frequencies based on the inverse relationship between oxidation of Fe^2+^ to Fe^3+^ and ultrasonic frequencies [161]. The synthesis of particles in the presence of different ligands has also been performed, giving rise to particles between 5 and 16 nm [162].

Vijayakumar et al. [163] used a similar route to synthesize IONPs. They proposed a mechanism stating that ultrasonic waves produce the vaporization of water and further pyrolyzation into H and OH radicals due to prolonged temperature and pressure, which leads to the formation of hydrogen (H_2_) and hydrogen peroxide (H_2_O_2_) from the reaction between H_2_ and hydroxyl radicals, respectively. Meanwhile, the same energy also breaks down iron acetate into Fe(II) ions. These Fe(II) ions are later oxidized to Fe(III) using H_2_O_2_ as oxidant and forming Fe_3_O_4_ by using OH radicals [163]. There are several studies showing the effect of surfactants on the particles. Mukh-Qasim et al. [164] used SDS as stabilizer to get around 8.5 nm amorphous but water dispersible Fe_3_O_4_ particles, while Rahamwati et al. used iron sands along with different concentrations of PEG-6000. This latter group showed that, as PEG concentrations increased, the crystallite size of the particles increased [165]. They also showed that the morphology of the particles shifted from flower-like to cubes to spheres with increasing PEG concentrations. Kim et al. [166] synthesized OA-capped IONPs, which form a ferrofluid when dispersed in chitosan, with a hydrodynamic diameter of 65 nm, thus being potential MRI CAs. This method can also be used to synthesize composite nanoparticles [167] or other ferrites [168, 169].This method has also been used for surface functionalization in very short time [170].

The main advantage of this method is its accelerated nature to produce nanoparticles with good yield, but it falls short when it comes to phase homogeneity.

### Microwave Synthesis

This is a modern-day hydrothermal method of synthesizing nanoparticles and one of the most used in recent days due to its much-improved kinetics of crystallization. It requires as low as 10 s and yields small and monodisperse particles due to homogenous heating [171].

Palchik et al. were among the first to use this method in a domestic microwave oven and suggested that the synthesis of particles was happening due to thermal breakdown of Fe(CO)_5_, which in turn was taking place due to heating of chlorobenzene, since Fe(CO)_5_ is a microwave resistant compound [172]. This indirectly marks the importance of the solvent. On the other hand, Liu et al*.* demonstrated the importance of water in maintaining a stable heating environment, along with the role of stoichiometry [173]. Another important parameter that have been studied extensively is the nature of the surfactant, with studies reporting the use of different concentrations of OA [174], amino acids [175], polyethylene glycol (PEG) [176], and different ratios of OA and oleylamine (OLA) [177]. OA is shown to increase saturation magnetization with increasing concentration, with no definite trend in size. However, concentrations beyond 0.35 mmol/dm^3^ led to agglomeration and the product became difficult to isolate. Recently, amino acids such as glycine have been shown to reduce the crystallite size of IONPs, opening the path to explore other amino acids [175]. The presence of PEG in the reaction has also been shown to lead to smaller IONPs, as compared to the reaction in its absence. When the reaction is performed in the presence of PEG, it tends to favor the formation of magnetite instead of maghemite. This happens due to PEG being sacrificial in nature and thus preventing oxidation. High microwave power and low synthesis time also favors the formation of maghemite [176]. Other studies have shown that the presence of OA during the synthesis, along with OLA, reduces aggregation among particles [177]. Temperature has also been shown to transform phases in IONPs [178]. Blanco-Andujar et al. proposed a facile method to synthesize citric acid coated IONPs and potentially scale them up [179]. The importance of aging temperature on crystallinity can be seen when Fe_2_O_3_ nanocubes are synthesized by decomposing iron oleate in a microwave and aging it in an autoclave at 180 ℃ for different time intervals [180]. Particles aged for 20 h showed cubic shape and higher saturation magnetization. Hu et al. [181] argued that the precursor is the most important parameter by synthesizing three phases of iron oxide, hematite, magnetite and maghemite, using FeCl_3_ alone or in combination with FeCl_2_.

Literature suggests that the morphology and composition of the particles can also be controlled using this method. Different morphologies, such as lamellar sheets [182], octahedrons [182] and hexagonal plates [183] are synthesized by slight changes in salts. Cu-doped IONPs with good colloidal stability are obtained in 10 min [184] using the microwave method. In fact, even using a domestic microwave, sizes of 8–10 nm can be easily achieved [185].

This method has been shown to be better than hydrothermal [186] or thermal decomposition [187] in terms of size, crystallinity and saturation magnetization. However, particles thus synthesized display lower surface reactivity than those synthesized using the thermal decomposition method, although with more ease of stabilization. The versatility of this method is acknowledged by its association with different methods: coprecipitation [179], thermal decomposition, [177] polyol [188] and sol–gel methods [189]. The particle size can be varied by modulating the power and hence the temperature, the time spent in the reactor, the cooling rate, etc. This method has become more popular recently due to its multiple advantages.

### Biosynthesis

This is an eco-friendly method as most of the constituents needed are available from nature directly or indirectly. It generally involves the use of microbes [190] or plant extracts [191] to synthesize nanoparticles. Lovely et al. [192] were the first to use a microbe, GS-15, to form magnetite nanoparticles. Thereafter, many different magnetic bacterial strains were found and studied in order to produce IONPs [193–197].These nanoparticles are formed by the reduction/hydrolyzing capabilities of these biological entities. However, when a bacterium is used, its nature as well as its incubation time becomes an important parameter since it allows changes in size and morphology [198, 199]. Even fungi such as *Fusarium oxysporum* and *Verticillium *sp., have been shown to possess hydrolyzing capabilities to form different sizes and shapes of nanoparticles [200]. Viruses such as tobacco mosaic virus (TMV) have also been used as templates to synthesize nanotubes [201]. Iron oxides formed by microbial reduction have been shown to lead to phase transformation with better crystallinity, although decreasing their reducibility [202].The scalability issue has also been answered by using a 30-l reactor, although there is only one report of such nature [203].

Plants or plants extracts have been used for the synthesis of nanoparticles [204]. Most recently, IONPs have been synthesized using figs, *Ficuscarica* and *Plantago major* extracts, which, apart from reducing precursors, also cap and stabilize the particles. These reactions have been concluded to take place due to the presence of phenols, and normally lead to sizes ranging from 2 to 50 nm [205, 206].

The main advantages of this method are that it is energy saving and non-toxic. Also, there is an unlimited supply of reducing agents, making it economically viable. On the other hand, its major disadvantage is unpredictability regarding the nature of the particles, with less control over the shape and size, along with uncertainty of yielding monodisperse particles when scaled up.

### Other Methods

Several different methods for the synthesis of IONPs have not been described above due to a dearth of information in the literature. Alvarez et al. [207] developed a novel flow injection synthesis (FIS) method to fabricate magnetite nanoparticles in a capillary reactor, and produced homogenous particles of 2–7 nm with high reproducibility. There have been reports of the use of metal rods as anodes and electrochemical deposition in the presence of surfactants to yield 3–8 nm particles [208–210]. Chemical vapor deposition (CVD) [211, 212] has been used to fabricate thin films and morphology-controlled nanoparticles. Other methods, such as synthesis in a reactor [213], the solution combustion method [214], and the use of microfluidic channels on a chip [215, 216], have also been introduced.

All the methods described above have their own pros and cons, and the choice of one or the other depends on the application for which the nanoparticles are being developed. Thus, for nanoparticles to be used as MRI CAs, the most suitable methods appear to be the thermal decomposition or microwave methods, since they provide a very narrow size distribution, high saturation magnetization and good morphology control.

## Functionalization of IONPs

One of the most important topics in the design of IONPs for in vivo applications is functionalization, which provides NPs with high stability in physiological media, stealth and vector targeting properties. In this section, we summarize the most relevant methods to functionalize IONPs for clinical purposes.

### Organic Supra-structures

In recent decades, a class of highly branched and monodispersed macromolecules with well-defined three-dimensional (3D) architectures, such as nanomicelles, dendrimers, liposomes and nanogels, have been developed to create hybrid nanoscale materials for imaging and therapeutic applications.

#### Nanomicelles

**Fig. 4:**
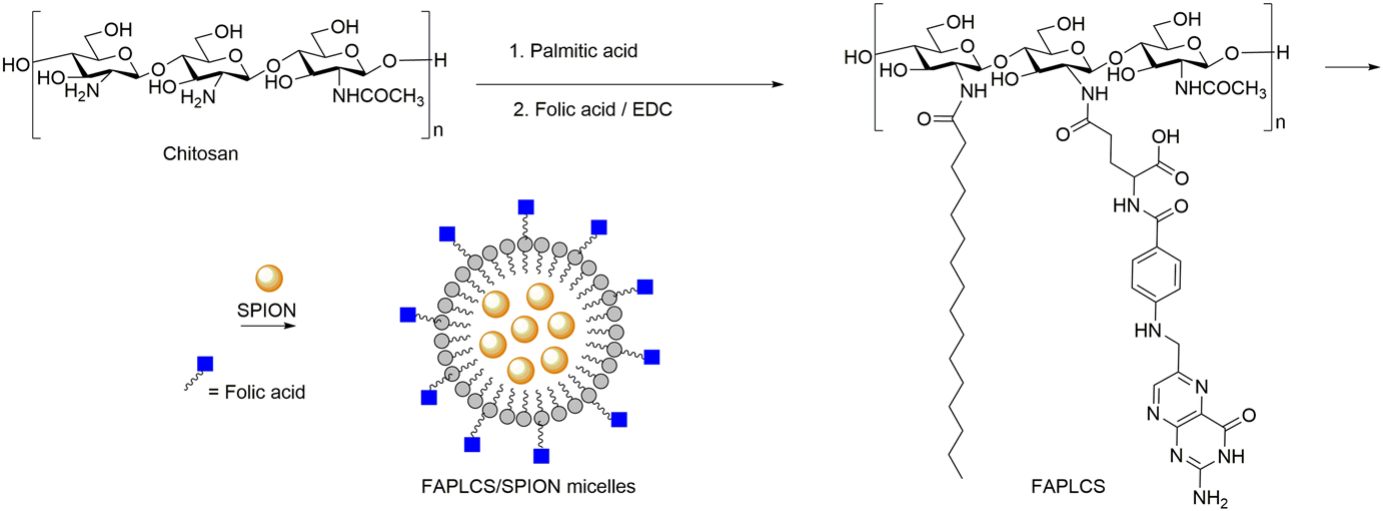
Synthesis of chitosan derivative polymeric micelles encapsulating superparamagnetic iron oxide nanoparticles (SPIONs) [308]

Nanomicelles are formed by the self-assembly of surfactant molecules or copolymers that adopt a core–shell like structure, thus entrapping in their inner core hydrophobic materials, such as drugs, dyes or inorganic nanoparticles (Fig. 4). The small size is another advantage of the micelles, which can be synthesized between 5 and 100 nm. This provides nanomicelles with long blood circulation times, which favor their active or passive accumulation in the target sites. Consequently, nanomicelles are generating great interest in the development of promising payload nanocarriers for theranostics [217–219]. Particularly interesting are the results obtained with hybrid nanosystems using polymer micelles loaded with IONPs. For instance, Jianping Bin and coworkers described the synthesis of a tumor-targeted MRI vehicle through the encapsulation of IONPs in self-aggregating polymeric folate-conjugated *N*-palmitoyl chitosan micelles [220]. In vitro and in vivo studies demonstrated the efficacy of folate-conjugated superparamagnetic iron oxide nanoparticle (SPION)-micelles in targeting and visualization by MRI of folate receptor overexpressed tumor cells. Torchilin et al. [221] created a diagnostic and therapeutic agent for in vivo use based on poly (ethylene glycol)-phosphatidylethanolamine (PEG-PE) micelles loaded with Paclitaxel (PTX), a poorly water soluble anticancer drug, and IONPs. The combination of both multi-modal cargos inside the micelles showed no property changes, either in the relaxivity of the IONPs or in the apoptotic anti-tumour activity of PTX.

#### Dendrimers

Dendrimers are a class of well-defined nanostructured macromolecules consisting of three critical architectural domains: the multivalent surface, the interior shells surrounding the core, and the core. These domains can be tailored for a specific purpose, such as a dendritic sensor [222, 223] or a payload carrier, the encapsulation of molecules in their interior shell being the most used application of dendrimers [224, 225]. One of the most common dendrimers is based on the chemical structure poly (amidoamine) (PAMAM), which has a large number of reactive amine groups on the periphery, making them an excellent platform to construct nanomaterials for biomedical applications [226–228]. Luong et al. [229] designed a promising theranostic agent based on the combination of IONPs and a hydrophobic anticancer drug loaded in a PAMAM dendrimer decorated with folic acid (FA). The design of this hybrid theranostic agent starts with functionalization of the SPIONs with activated carboxyl groups that bind folic acid-PAMAM dendrimers. The engineered SPIONs@FA-PAMAM showed great potential as MRI diagnostic agents, with increased internalization in cancer cells and better image contrast. Moreover, the encapsulation of hydrophobic anticancer drugs, such as 3,4-difluorobenzylidene-curcumin (CDF), in the dendrimers of the SPIONs@FA-PAMAM, enhances their anticancer activity by delivering a higher dose of CDF with high specificity to target cancer cells expressing folate receptors.

Dendrimers could also be used in gene therapy as gene delivery platforms. Xiao et al. [220] synthesized a nanohybrid dendrimer based on the combination of PAMAM dendrimers and IONPs through electrostatic interactions. First, the IONPs were functionalized with negatively charged polystyrene sulfonate (PSS), and then positively charged PAMAM dendrimers decorated with plasmid DNA were deposited onto the PSS-functionalized NPs, resulting in a nanohybrid material, PAMAM dendrimer/pDNA-coated MNPs. The results demonstrated that the efficiency of this hybrid system to transfect NIH 3T3 cells is strongly dependent on the dendrimer generation, the amine/phosphate groups ratio and the plasmid DNA concentration.

#### Liposomes

Liposomes comprise a lipid bilayer surrounding an aqueous core. They can be made from different lipid formulation and present different sizes depending on the method of preparation. Similarly to the organic macro-structures mentioned above, liposomes are able to encapsulate payloads in their hydrophobic or hydrophilic inner, which makes them excellent nanocarriers for therapeutic and imaging applications. Liposomes based on phospholipids are the most common vesicles for in vivo applications due to their great advantages, such as biocompatibility, biodegradability and reduced toxicity [230–232]. The incorporation of IONPs into liposomes is gaining increased attention of researchers as a way to synthetize more effective magnetic nanocarriers for in vivo applications. Di Corato et al. [233] designed a liposome formulation based on phosphatidylcholine lipids that entraps magnetic NPs and a photosensitizer in its interior. In a single synthesis method, higher concentrations of hydrophilic IONPs were encapsulated in the core, and a hydrophobic photosynthesizer, Temoporfin (marketed as Foscan), was incorporated into the lipid bilayer. The resulting magnetic liposome presented double functionality, magnetic hyperthermia and photodynamic therapy, which led to complete death of cancer cells in vitro and total ablation of solid-tumor in vivo.

Zheng et al. [234] synthetized a tumor-specific peptide-decorated liposome containing payloads of IONPs and an anti-cancer drug in their inner core and lipid bilayer, respectively. Like the protocol described above, the combination in a single pot reaction of egg phosphatidylcholine, cholesterol, paclitaxel (PTX), different 1,2-distearoyl-sn-glycero-3-phosphoethanolamine (DSPE) phospholipids, such as DSPE-PEG and cell penentrating peptide-modified DSPE-PEG, and hydrophilic SPION, generated a theranostic liposome. The results confirmed the effectiveness for tumor targeting and anti-tumor activity through MRI in vivo experiments.

#### Nanogels

Nanogels (NGs) are nanosized water-soluble particles formed by crosslinked polymer networks with loading capacity of therapeutics. Stimuli-responsive NGs are a class of smart particles that respond to external physical changes, such as pH, temperature or redox agents [235, 236]. This behavior allows the controlled-release of payloads from NGs, minimizing possible side effects and avoiding the use of high doses. NGs can also be loaded with diagnostic agents, such as magnetic NPs, enabling their visualization and follow-up by MRI. These characteristics, together with the ease of uptake by cancer cells and tumor tissues due to their softness and fluidity, make NG-based nanosystems a high potential theranostic material [237, 238].

Qian et al. [239] prepared a hybrid NG system based on a thermo-responsive co-polymer [*N*-isopropylacrylamide, methacrylic acid and poly (ethylene glycol) methacrylate] that stabilizes hydrophobic IONPs and 10-hydroxy camptothecin (HCPT) in its inner compartment. The obtained IONP/HCPT-NG generated an increase in reactive oxygen species (ROS), allowed the enrichment of NG at the tumor site by applying an external magnetic field, and offered the possibility of being used as nanocarrier for photothermal therapy due to its absorption in the near infrared (NIR) range. In vivo results demonstrated that the combination of PTT and chemotherapy with external magnetic fields on IONP/HCPT-NGs, reduced the growth of primary tumors and prevented metastasis [239].

**Fig. 5:**
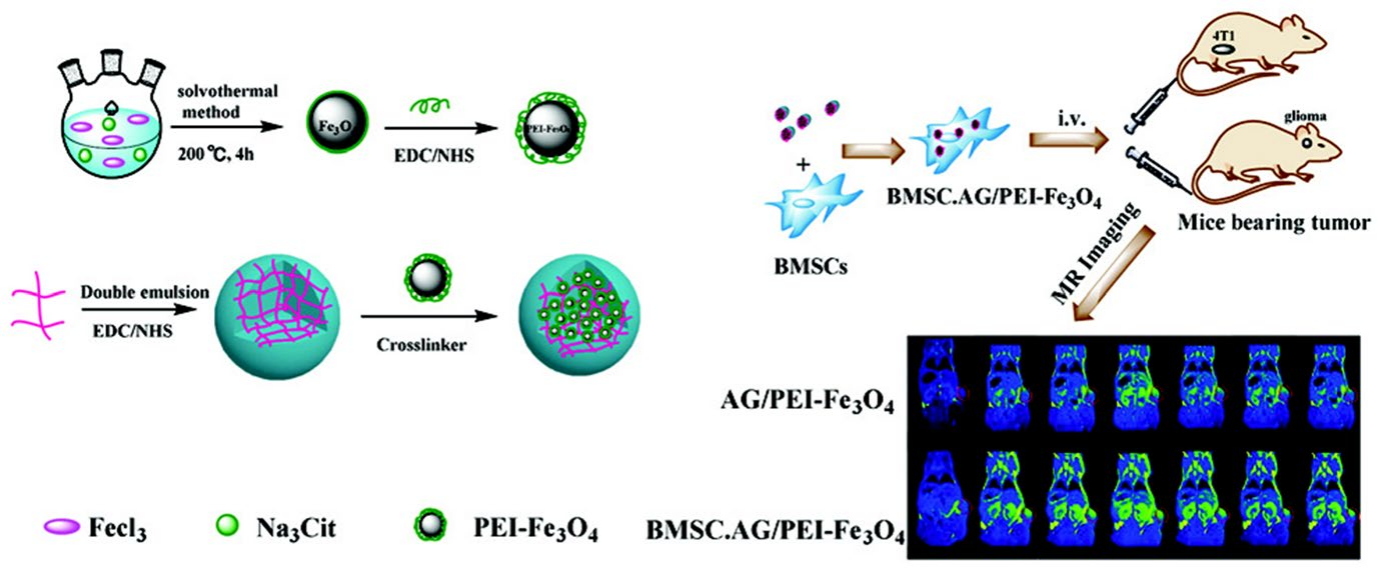
Schematic illustration of alginate/polyethilenimine-iron (III) oxide (AG/PEI-Fe_3_O_4_) and stem-cell-mediated delivery of nanogels (NGs) for enhanced breast or glioma tumor molecular resonance (MR) imaging. Reprinted with permission from [241]. Copyright (2019) Royal Society of Chemistry

Alginate (AG) is a natural polysaccharide that has been gaining attraction in recent years for the synthesis of polymeric nanomaterials with biomedical applications thanks to its biocompatibility, biodegradability and ease of gelation [240]. For instance, Hao et al. [241] designed alginate NGs loaded with IONPs and bone mesenchymal stem cells (BMSCs) for enhanced tumour MR imaging (Fig. 5). The potential advantage of using BMSCs as tumor delivery vehicles is that they are not tumorigenic and minimally immunogenic. In this way, polyethilenimine (PEI)-functionalized IONPs were crosslinked to AG NGs previously synthesized by a double emulsion method. The resulting AG/PEI-NP NGs were taken up by BMSCs without affecting cell characteristics. BSMC-AG/PEI-NP NGs were then used successfully for the in vivo diagnosis of different tumor models [241].

### Inorganic Coverage

Mesoporous silica is the most important inorganic coating material for IONPs due to the ease of functionalization, high stability, and vast surface area and pore volume to host large number molecules. These characteristics make hybrid mesoporous silica-IONPs excellent nanocarriers for controlled drug release therapies [242, 243].

**Fig. 6:**
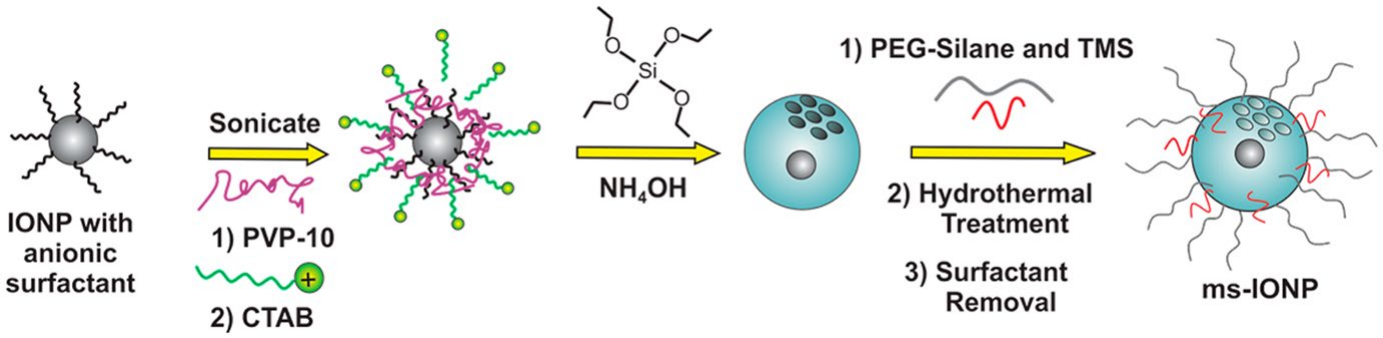
Synthesis of mesoporous silica-coated (ms)-IONPs. Polyvinylpyrrolidone (PVP)-10 was added to IONPs prior to cetyltrimethylammonium bromide (CTAB) addition and silica condensation to allow for CTAB colocalization with IONPs and to maintain a spacer layer between the silica shell and IONP core. Reprinted with permission from [245]. Copyright (2016) American Chemical Society

Based on this, Vallet-Regí et al. [244] designed a responsive silica matrix nanocarrier for tumor therapy based on magnetic NPs that combine the heat release mediated by magnetic hyperthermia and doxorubicin release through a thermo-responsive polymer. The as-prepared OA-capped IONPs are transferred into aqueous solution with CTAB, which helps the growth of the silica matrix by addition of tetraethyl orthosilicate (TEOS) as a silica precursor. Then, the silica-matrix-coated IONPs are functionalized with a methacrylate molecule as a polymer precursor to perform, using *N*-isopropylacrylamide (NIPAM), *N*-(hydroxymethyl)acrylamide (NHMA), and *N*,*N*′-methylenebis(acrylamide) (MBA) monomers, the synthesis of a thermoresponsive polymer surrounding the mesoporous silica-coated IONP (Fig. 6). Direct injection into the tumor site of Doxo-loaded mesoporous silica NPs, together with the application of amplified magnetic fields, provoked a synergistic effect between magnetic hyperthermia and chemotherapy that led to significant tumor growth inhibition and low toxicity [244].

Hurley et al. [245] demonstrated that the inclusion of functionalized mesoporous silica coating in IONPs cores results in stable NPs with high heat capacity and high MRI contrast. The anionic surfactants capped IONPs (a commercially available IONP called EMG-308) required pre-functionalization with polyvinylpyrrolidone (PVP) prior to silica condensation with the TEOS precursor. Finally, the functionalization with PEG and trimethyl silane derivates yields colloidal stable NPs with the same magnetic character that un-functionalized IONPs and minimal toxicity toward human skin fibroblasts. Furthermore, a direct injection into LNCaP prostate cancer tumours implanted in nude mice showed that these hybrid mesoporous silica-IONPs can improve the heating and imaging contrast of IONPs [245].

### Ligand Exchange

Ligand exchange is a very complicated coating strategy that involves multiple interactions potencials/forces. It requires the use of reactive binding molecules that enable the replacement of capping agents attached to the nanoparticle surfaces. This binding between the iron atoms of the IONP and the anchor group of the ligand molecules is mediated by electrostatic interactions. Therefore, the nature of the anchor group is determinant in the search for highly stable ligand molecules at the IONP surfaces. In addition to anchor groups, the hydrophilic balance of the ligand is also important to render water-soluble NPs [236, 246, 247]. In our group, we have developed different ligand formulations to functionalize IONPs to obtain soluble and stable NPs in physiological media for in vivo MRI applications. These ligands are based on a gallol group as a strong binder and PEG chains as hydrophilic tunable spacers, which also minimize plasma protein adsorption. In this manner, we have demonstrated that selection of the right molecular weight of PEG chain and the outermost charged group of the ligand plays a fundamental role in the fate and bioavailability of intravenously injected IONPs. Thus, a ligand with a PEG chain between 1500 and 3000 Da and neutral outermost groups showed the best stealth properties, resulting in longer blood circulation times and higher bioavailability without increased toxicity [248–250].

## Applications of IONPs in MRI

Among the main clinical diagnostic techniques, MRI stands out for its unique combination of qualities, such as its non-invasive character, the absence of ionizing radiation, excellent image quality, and its ability to provide both anatomical and functional information [251]. The MRI signal comes mainly from the protons of the water molecules, while the image contrast is generated from differences in the intensity of this signal among different tissues, which depends on the concentration, relaxation times (T_1_ and T_2_) and mobility of the water molecules within each tissue [252, 253]. Additionally, image contrast can be further enhanced using CAs. Although there are several mechanisms that can produce MRI contrast, such as chemical exchange saturation transfer (CEST) or hyperpolarization, most MRI CAs produce contrast by altering the relaxation times of the surrounding water protons [254, 255]. The capacity of a CA to decrease the relaxation times (T_1_ or T_2_) is given by a parameter known as relaxivity (r_1_ or r_2_), which is expressed in mM^−1^·s^−1^.

In MRI, among the most commonly used CA are chelates of paramagnetic gadolinium(III) ions (Gd^3+^). However, conventional Gd-chelates have some important limitations, such as the lack of diagnostic specificity and the toxicity associated with their use as a result of the unexpected release of free Gd ions [256, 257]. Magnetic NPs have emerged as a promising alternative to overcome these limitations [258].

### IONPs in Tumor Diagnosis

#### Untargeted IONPs

**Fig. 7:**
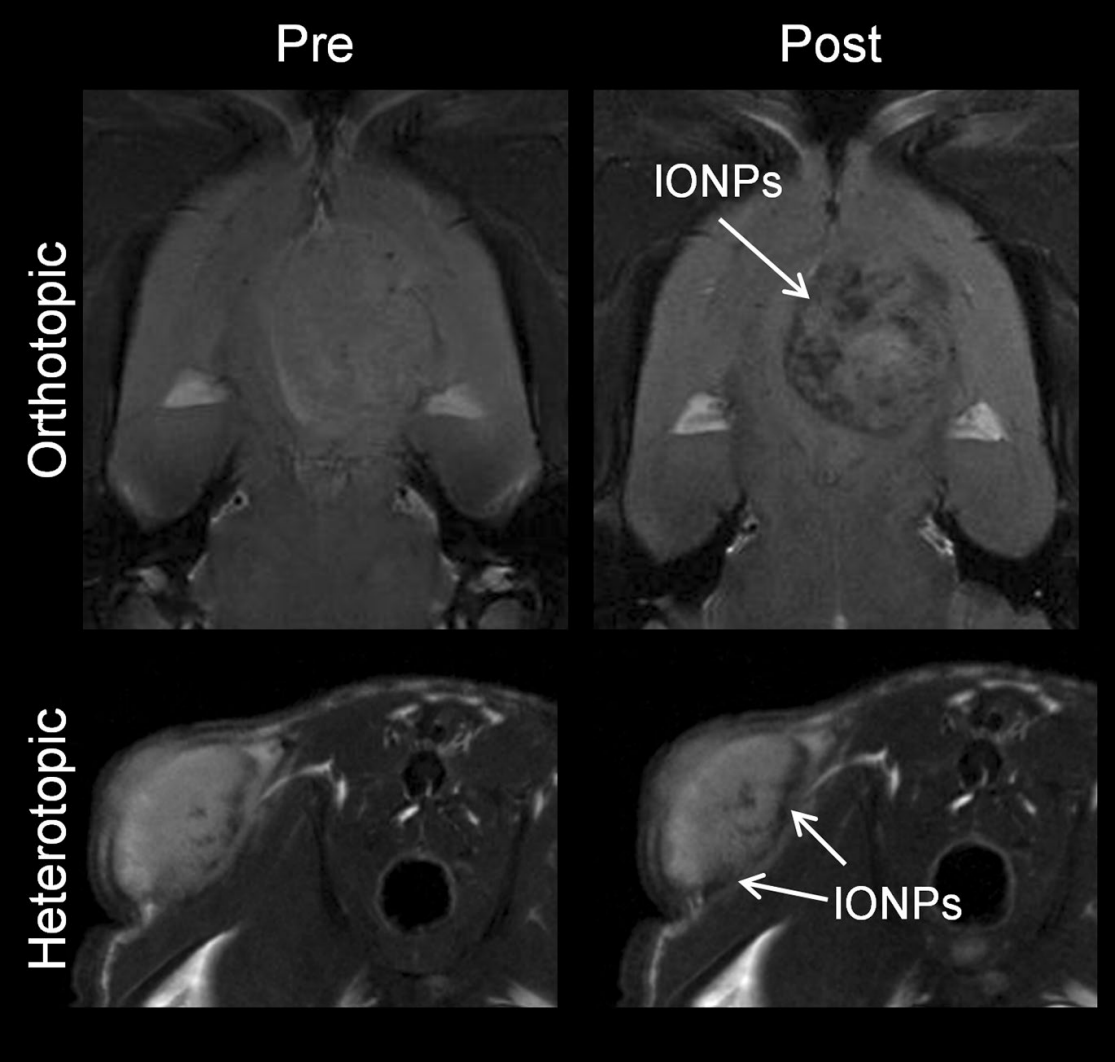
C6 brain tumor model implanted orthotopically (upper panels) and heterotopically (lower panels). Left) T_2_-weighted MR images before the injection of IONPs; right) T_2_-weighted MR images 1 h after the injection of IONPs

The evaluation of IONPs as CAs in cancer research is performed mainly in rodent models, called ‘indirect xenografts’ [259]. Cancer cells can be implanted either into a tissue unrelated to the original tumor site (heterotopic model) or into the corresponding anatomical position (orthotopic model) [260] (Fig. 7). The route of administration of magnetic NPs is also relevant as it influences the biodistribution and pharmacokinetics of the CA. Several administration routes have been used in preclinical studies, mainly intratumoral, intraperitoneal or intravenous injection; for obvious reasons, the latter is the most interesting for clinical applications. After intravenous administration, IONPs have been described to accumulate in tumors due to the EPR (Enhanced Permeability and Retention) effect. This passive transport is determined by the high vascularization of tumors, and therefore increased blood flow, together with increased vascular permeability and poor lymphatic drainage [261]. Efremova et al. [262] developed IONPs for diagnosis of breast cancer in a heterotopic model. They observed that IONPs accumulated passively inside the tumor 24 h after intravenous injection using T_2_-weighted MR images. Similar studies have been conducted using orthotopic models of breast cancer [263, 264], pancreatic cancer [265] and glioblastoma multiforme (GBM) [266]. All these studies conclude that IONPs accumulated in the tumor due to the EPR effect; however, most of them lack quantitative analyses, which are necessary to determine the amount of IONPs that actually reach the tumor.

Intratumoral administration could be an alternative for tumor therapy when the CA is not able to reach the tumor by a venous route. However, this approach has serious limitations for diagnostic applications since, in most cases, it would not add any useful information to that already provided by the MR images without CA. Furthermore, intratumoral administration makes no sense when it comes to very early diagnosis, detection of metastasis or in the case of inaccessible tumors. Nevertheless, several preclinical studies have been conducted using intratumoral injection of IONPs [267–269]. The authors used qualitative MRI to evaluate the distribution of IONPs throughout the tumor, which showed that IONPs spread slowly and inefficiently. Therefore, in these studies the information provided by MRI after the intratumoral injection of IONPs serves as proof of concept, but, as we have just mentioned, it is of no practical value for potential clinical applications.

In conclusion, up to now, untargeted IONPs have not proven to be a good alternative to conventional MRI CAs for cancer diagnosis.

#### Targeted IONPs

**Fig. 8:**
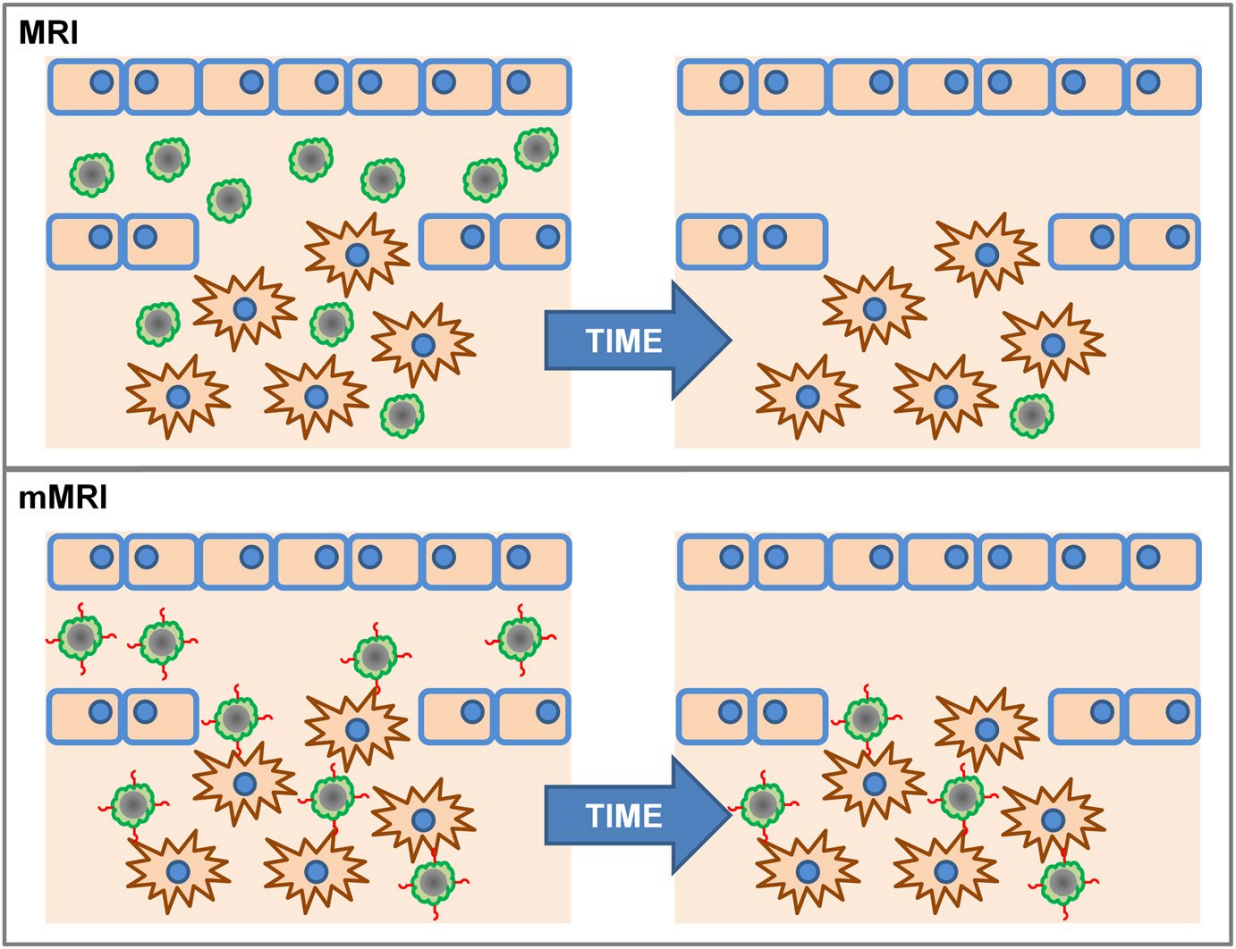
Scheme of the non-targeted (top) and targeted IONPs (bottom)

To improve the accumulation of IONPs in tumors, a promising strategy is conjugation with targeting segments [5]. In principle, this functionalization would allow not only the visualization of IONPs by MRI, but would also offer the possibility of visualizing cellular and subcellular functions and processes in living organisms without perturbing them, giving rise to so-called molecular MRI (mMRI) [270], which was first described by Richard Klausner [271, 272] (Fig. 8).

It is worth mentioning that the targeted strategy for cancer diagnosis was described before the untargeted strategy. Reimer et al. [273] described in 1990 the diagnosis of liver cancer after the intravenous administration of IONPs-arabinogalactan conjugates, in both heterotopic and orthotopic models.

IONPs have been functionalized with epithelial growth factor (EGFR) antibodies for the diagnosis of breast cancer [274], pancreatic/stomach cancer [275] and brain cancer [276]. Because there is an established relationship between mutations involving overexpression or overactivity of EGFR and various types of cancer, this receptor is currently one of the most important targets in cancer research [277–279]. Similarly, PSCA (prostate stem cell antigen) antibody was bound to IONPs for diagnosis of prostate cancer [280].

Integrins receptor, especially α_5_β_3_, has been found to be differentially overexpressed in tumors, playing a vital role in tumor angiogenesis [281–283]. Integrins are recognized mainly by short peptide sequences, such as Arg–Gly–Asp (RGD). Therefore, some NPs functionalized with RGD have been proposed for the diagnosis of brain cancer [284], colon cancer [285] or fibrosarcoma [286], among others.

Among other functionalization molecules for targeted diagnosis, it is worth highlighting the use of aptamers for kidney [287] and liver cancer [288], peptides for prostate and liver cancer [289, 290], and flavin adenine dinucleotide (FAD) for prostate cancer [291].

**Fig. 9:**
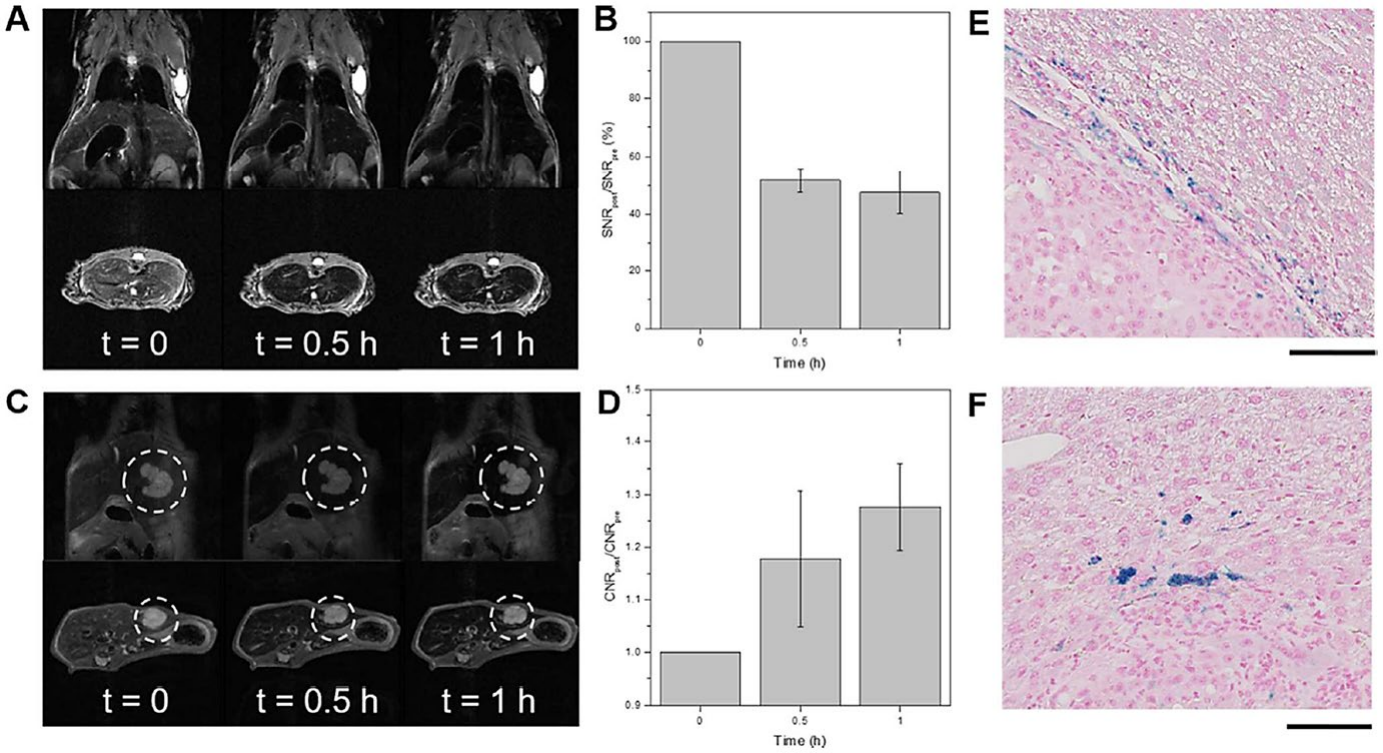
**a** In vivo MR images of a NCr nude mouse at different time points after intravenous injection of IONPs. **b** Quantification of liver contrast collected at different time points after accumulation of IONPs in NCr nude mice. **c** In vivo MR images of liver tumor orthotopic xenographs at different time points after intravenous injection of IONPs. **d** Quantification of contrast-to-noise ratio (CNR) of tumor-to-liver contrast at different time points. **e**, **f** Histopathological analysis of mouse liver 1 h after the intravenous injection of IONPs. Reprinted with permission from [292]. Copyright (2018) American Chemical Society

Recently, Chee et al. published an interesting study in which they described the design of a library of short peptides and ligands to functionalize IONPs. From this library, they selected the ligand that provided IONPs with the best characteristics for in vivo use, namely, long term stability, non-specific binding to live cells and absence of cytotoxicity at high concentrations. IONPs functionalized with this ligand showed a significant increase in contrast between the liver tumor and the healthy liver tissue, as compared with commercial MRI CAs [292] (Fig. 9).

Finally, it is worth mentioning that, in clinical diagnosis, positive contrast is generally preferred over negative contrast because it avoids the potential confusion of signal decay caused by negative CAs with signal voids caused by magnetic field inhomogeneities induced by air, metal prosthesis, etc. Thus, a very recent study described the use of Cu as a dopant agent that enhances the positive contrast of IONPs functionalized with RGD for targeted diagnosis of breast cancer [184]. Even though many IONPs show dual contrast potential, that is, r_2_/r_1_ ratio  between 3 and 10, their use in vivo as positive CAs is limited by the acquisition conditions of conventional T_1_-weighted MRI sequences, which are usually based on the spin-echo acquisition scheme and therefore require relatively long echo times. However, the introduction of new MRI acquisition sequences, such as ultra-short echo time (UTE) sequences, is making it possible to detect IONPs as positive contrast [293].

### IONPs as CA in Other Pathologies

Although most research in IONPs designed to serve as MRI CAs is focused on cancer diagnosis, there are many other pathologies that can benefit from advances in this field of research, as discussed henceforth. Recent investigations have demonstrated that an acetylcholine-sensitive mMRI nanosensor can be used for measuring the endogenous release of acetylcholine in the rat brain after its intracerebral administration [294]. Similarly, after intracerebral administration of alginate-coated IONPs, changes in Ca^2+^ levels have been monitored following a quinolinic acid-induced striatal lesion [295]. Other magnetic nanostructures have been used for the detection of brain inflammation [296]. These particular nanostructures are based on IONPs coupled covalently through peptide linkers that have been designed to be cleaved by the intracellular macrophage cathepsin, which results in microparticles of iron oxide (MPIO) and allows the fate of magnetic NPs to be tracked. This is because the MPIO, once sequestered by macrophages in the liver, decrease their relaxivity, while particles that associate with their target tissue in the brain remain unaltered and functional.

Thrombosis is a major clinical problem whose incidence has not decreased over the last 20 years and is involved in several pathological disorders such as myocardial infarction, ischemic stroke or pulmonary embolism, among others [297]. Early detection is essential for effective treatment, but it remains challenging in practice. P-selectin is an adhesion molecule, overexpressed at the surface of endothelial cells and platelets upon activation, which plays a fundamental role in thrombus formation [298]. Based on this fact, Suzuki et al. [299] innovated a fucoidan (a natural sulfated polysaccharide with high affinity for activated platelets through P-selectin)-coated USPIONs to visualize by MRI arterial thrombi in the early stage of the disease. Other investigations used PLGA-coated IONs, functionalized with EWVDV peptide, which has a high affinity and specificity for P-selectin, to target thrombi for both diagnosis and treatment through the induction of thrombolysis [300].

### Other Applications

IONPs have also been used in combination with MRI for many other in vivo applications, such as imaging of activated microglia during brain inflammation [301], tracking of stem cells [302–304], image-guided treatment of anemia using bacteria loaded with IONPs [305], or to carry out vascular imaging [306, 307], among others.

## Conclusions

Recent advances in nanotechnology applied to biomedical research have made possible the development of a new generation of magnetic nanomaterials with great potential as MRI CAs. IONPs stand out due to their excellent combination of properties for in vivo applications, that is, their superparamagnetism along with their high biocompatibility. Also, advanced functionalization strategies have allowed these IONPs to be specifically targeted to different tissues or cells to perform molecular imaging. However, in spite of all these advances, and the large number of studies carried out in this field, very little clinical translation has been achieved so far. The main reasons behind this relative failure are very likely related to reproducibility and scalability issues during the synthesis process, which must be further improved. Also, in vivo studies must be thoroughly designed to include comprehensive toxicity assays and preclinical imaging studies using appropriate animal models.
