# Correction to: Magnetic Nanoparticles as MRI Contrast Agents

**DOI:** 10.1007/s41061-021-00340-y

**Published:** 2021-06-14

**Authors:** Ashish Avasthi, Carlos Caro, Esther Pozo-Torres, Manuel Pernia Leal, María Luisa García-Martín

**Affiliations:** 1 BIONAND - Centro Andaluz de Nanomedicina y Biotecnología, Junta de Andalucía-Universidad de Málaga, C/Severo Ochoa, 35, 29590 Málaga, Spain; 2 Departamento de Química Orgánica y Farmacéutica, Facultad de Farmacia, Universidad de Sevilla, 41012 Seville, Spain; 3 Networking Research Center on Bioengineering, Biomaterials and Nanomedicine, CIBER-BBN, Málaga, Spain

## Correction to: Topics in Current Chemistry (2020) 378:40 10.1007/s41061-020-00302-w

The article Magnetic Nanoparticles as MRI Contrast Agents, written by Ashish Avasthi, Carlos Caro, Esther Pozo-Torres, Manuel Pernia Leal and María Luisa García-Martín was originally published Online First without open access. After publication in volume 378, issue 3, page 40, the Authors requested that the article be Open Choice to make the article an open access publication on May 20th.

This article is licensed under a Creative Commons Attribution 4.0 International License, which permits use, sharing, adaptation, distribution and reproduction in any medium or format, as long as you give appropriate credit to the original author(s) and the source, provide a link to the Creative Commons license, and indicate if changes were made. The images or other third-party material in this article are included in the article’s Creative Commons license, unless indicated otherwise in a credit line to the material. If material is not included in the article’s Creative Commons license and your intended use is not permitted by statutory regulation or exceeds the permitted use, you will need to obtain permission directly from the copyright holder. To view a copy of this license, visit https://creativecommons.org/licenses/by/4.0/.

The original article has been corrected.

**Open Access** This article is licensed under a Creative Commons Attribution 4.0 International License, which permits use, sharing, adaptation, distribution and reproduction in any medium or format, as long as you give appropriate credit to the original author(s) and the source, provide a link to the Creative Commons licence, and indicate if changes were made. The images or other third party material in this article are included in the article’s Creative Commons licence, unless indicated otherwise in a credit line to the material. If material is not included in the article’s Creative Commons licence and your intended use is not permitted by statutory regulation or exceeds the permitted use, you will need to obtain permission directly from the copyright holder. To view a copy of this licence, visit http://creativecommons.org/licenses/by/4.0/

